# Anterior Cruciate Ligament Ganglion Cyst and Mucoid Degeneration: A Review

**DOI:** 10.7759/cureus.1682

**Published:** 2017-09-13

**Authors:** Raju Vaishya, Abdulrazaq Esin Issa, Amit Kumar Agarwal, Vipul Vijay

**Affiliations:** 1 Department of Orthopedics, Indraprastha Apollo Hospital, New Delhi; 2 Orthopaedics, GOMBE State University

**Keywords:** anterior cruciate ligament ganglion cyst, mucoid degeneration

## Abstract

Mucoid degeneration and ganglion cyst are two distinct non-traumatic lesions of the anterior cruciate ligament (ACL) that most commonly occur discretely but occasionally coexist. They are relatively uncommon, and their exact aetiopathogenesis is still not clear. ACL ganglion cyst occurs more frequently among relatively younger patients compared to mucoid degeneration. They could be asymptomatic and discovered incidentally while evaluating the knee for other pathologies. Symptomatic cases of the two conditions present with nonspecific chronic pain, painful limitation of terminal flexion and extension, and mechanical block (among other symptoms), similar to that of other internal derangement pathologies of the knee. Magnetic resonance imaging is the investigation of choice, and diagnostic criteria are defined. Arthroscopic management of these conditions efficiently provides an improvement in symptoms without instability. Computed tomography scan guided aspiration is also useful in selected cases of ACL ganglion cyst. ACL ganglion cyst and mucoid degeneration should be considered in the differential diagnosis of chronic knee pain and stiffness of unspecified etiology.

## Introduction and background

Anterior cruciate ligament (ACL) ganglion cyst and ACL mucoid degeneration are two non-traumatic pathologies that have been increasingly found be associated with insidious onset chronic knee pain. They may also present as a limited range of motion of the knee due to either pain or mechanical block [1-4]. ACL ganglion cyst and ACL mucoid degeneration most often occur independently, but they may also coexist [5]. The pathogenesis of these two lesions remained controversial but suggested theories include synovial tissue herniation, post-traumatic mucoid degeneration, ectopic synovial tissue theory, and mucoid degeneration of connective tissue [6-7]. There are no fixed clusters of symptoms that are pathognomonic of this condition. These are often discovered incidentally on magnetic resonance imaging (MRI) of the knee or knee arthroscopy while evaluating symptoms of knee pain and limitation of knee motion. Awareness and a high index of suspicion are required to diagnose these conditions. The gold standard investigation for diagnosing an ACL ganglion cyst and mucoid degeneration is MRI [8-9]. Excellent results have been reported following arthroscopic treatment with complete resolution of symptoms [10].The purpose of this review article is to provide an overview of the diagnosis and treatment of ACL ganglion cyst and ACL mucoid degeneration.

## Review

Anterior cruciate ligament ganglion cyst

A ganglion is a cyst containing a mucin rich fluid bounded by pseudomembrane and are usually associated with a joint or tendon sheath. Superficial ganglion cyst in the wrist, foot, and knee are palpable and easily diagnosed clinically, but deeper ganglia such as intra-articular knee ganglia, suprascapular notch ganglia, and periacetabular ganglia are difficult to diagnose clinically as they are not palpable. The reported incidence of intra-articular ganglia cyst of the knee ranged from 0.20% - 1.33% on knee MRI and 0.6% - 2.0% on knee arthroscopy [11-12]. Almost 62% of them are located on the ACL. ACL ganglion cyst coexists with ACL mucoid degeneration in about 35% of cases. With the increasing awareness of ACL ganglion cyst, several cases have been reported in the literature in the recent past. The exact pathogenesis of ACL ganglion cyst is still controversial. Theories proposed include post traumatic mucinous degeneration of connective tissue mediated by the local release of hyaluronic acid, displacement of synovial tissue during embryogenesis, and herniation of synovium into a defect of the surrounding tissue. The mean age of patients with discrete ACL ganglion has been quoted as 39 years old (age range of 19-60) [13], although isolated cases in children ages two years old [13-14], nine years old [15], and 12 years old [16] have been reported in the literature. Male preponderance has been reported [17].

Clinical Features  

There are no fixed clusters of symptoms that are diagnostic of ACL ganglion cyst. They should be suspected in patients with chronic knee pain and limitations in range of motion. Isolated ACL ganglion that is solely responsible for knee symptoms without any concomitant intra-articular pathology is regarded as ‘symptomatic', while incidentally detected ACL ganglion cysts associated with other knee lesions, and obviously not responsible for the clinically dominant symptoms, are classified as ‘asymptomatic'. The most common presentation is chronic knee pain of insidious onset, worsened by extreme knee movements. They present as a variable duration of symptoms from weeks-to-months and sometimes years [18]. Mechanical locking, clicking sensation, and stiffness also occur frequently. A ganglion cyst that is located anteriorly at the tibial attachment results in extension block while those located posteriorly produce flexion block [19-20]. Usually, there is no history of knee instability. These symptoms are mostly of spontaneous onset without a history of trauma. When trauma is reported it's usually trivial and of little significance. The clinical examination may reveal knee joint effusion [21], joint line tenderness [22-23], wasting, and limitation of motions. The ACL stability tests, such as the anterior drawer test, Lachman test, and pivot shift tests, are negative.

Investigations

**Figure 1:**
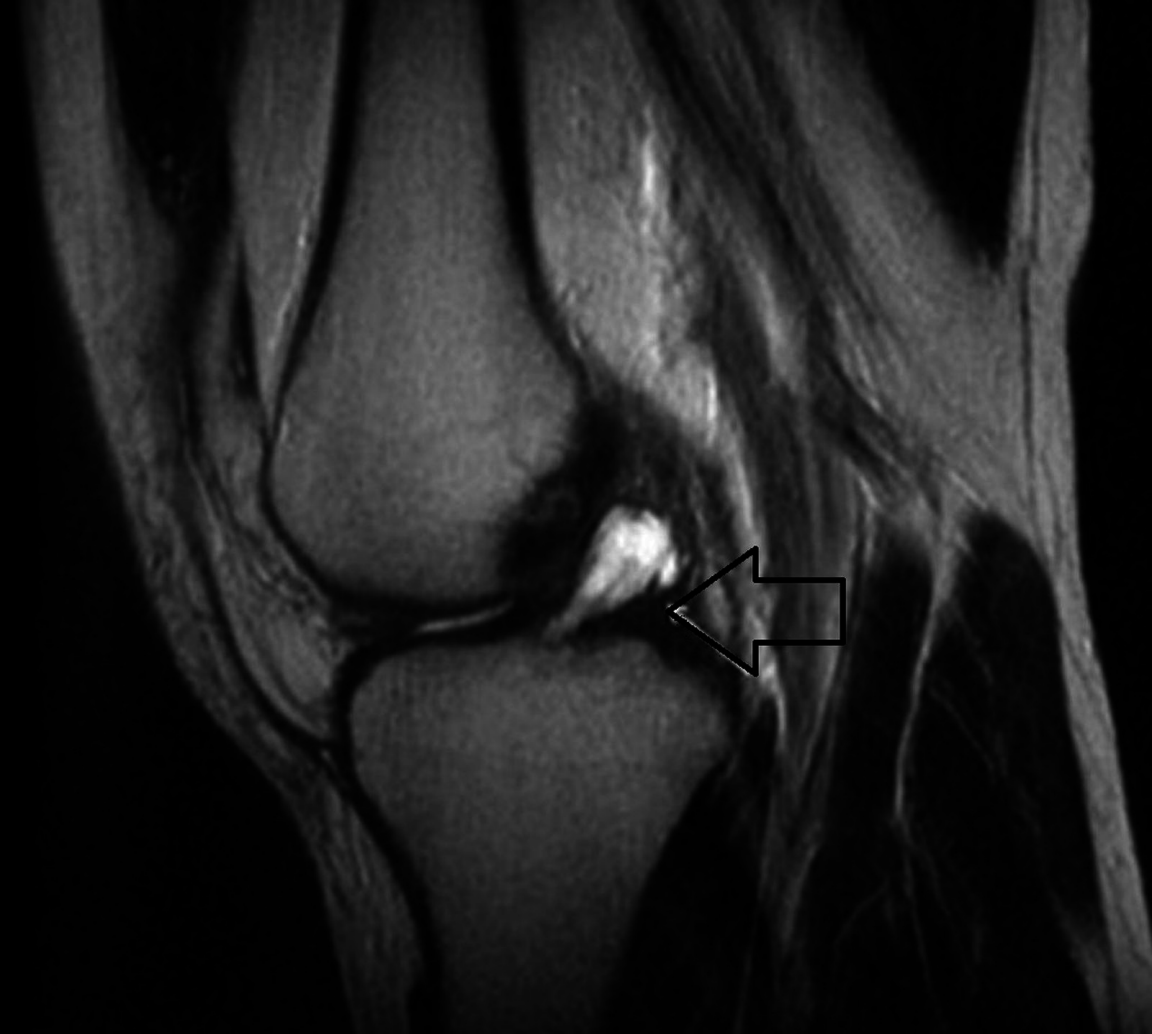
Sagittal T2-weighted magnetic resonance image showing fusiform mass (arrow) that is almost homogeneously hyperintense in the anterior cruciate ligament (ACL).

**Figure 2:**
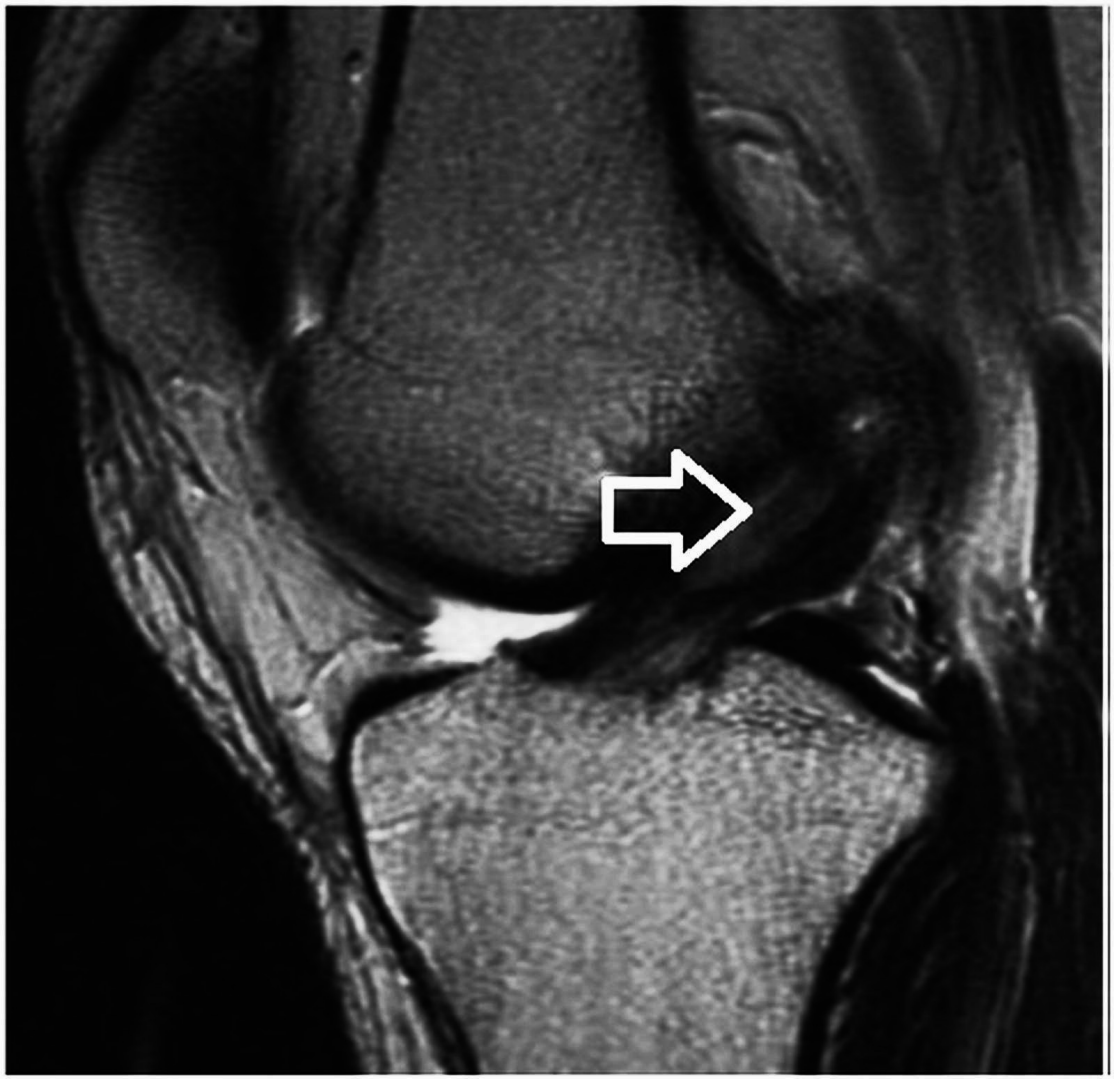
Sagittal T2-weighted magnetic resonance image, which shows intact ACL fibers with ganglion cyst (arrow). ACL: anterior cruciate ligament

Conventional X-rays do not show any specific features of ACL ganglion cyst. Computed tomography (CT) scan and arthrography are also specific but are of low diagnostic value. MRI scan is the gold standard investigation because of its multiplanar capability, superior identification of anatomic and morphologic interpretation of synovial tissue relative to other structures, and ability to detect other intra-articular pathologies. It is sensitive, specific, noninvasive, and useful in planning operative treatment. An ACL ganglion cyst appears as fusiform, or rounded, with a clear boundary extending along the course of the ACL or interspersed within the fiber of the ligament. ACL ganglion cyst exhibits hypointense signals on T1 weighted images (Figure 1) and hyperintense signals on T2-weighted images (Figure 2).

Bergin’s MRI diagnostic criteria for ACL ganglion cyst were a fluid signal in the substance of the ligament having at least two of the three criteria: (a) mass effect of ACL fibers, (b) lobulated margins, and (c) ACL fluid disproportionate to the joint. The ACL has to be intact from tibial to femoral insertion to exclude partial or complete ACL rupture (Table 1). Associated internal derangement, like meniscal tear and articular cartilage damage, occurs in 22% - 50% of patients [24].

**Table 1:** Bergin’s MRI Criteria for Anterior Cruciate Ligament Ganglion Cyst and Mucoid Cyst Degeneration ACL: anterior cruciate ligament, MRI: magnetic resonance imaging

	Ganglion cyst	Common criteria	Mucoid degeneration
1	Fluid signal in the substance of the ligament with at least two of the three following criteria	ACL fibers intact and uninterrupted from tibial to femoral insertion	Ligament fibers poorly seen on T1-weighted images
2	Mass effect on anterior cruciate better ligament fibers	Possibly associated with joint effusion or bony cysts	Ligament bundles and fibers seen on T2-weighted images
3	Ligament signal stronger than joint fluid		
4	Lobulated with definite margins		

Treatment

**Figure 3:**
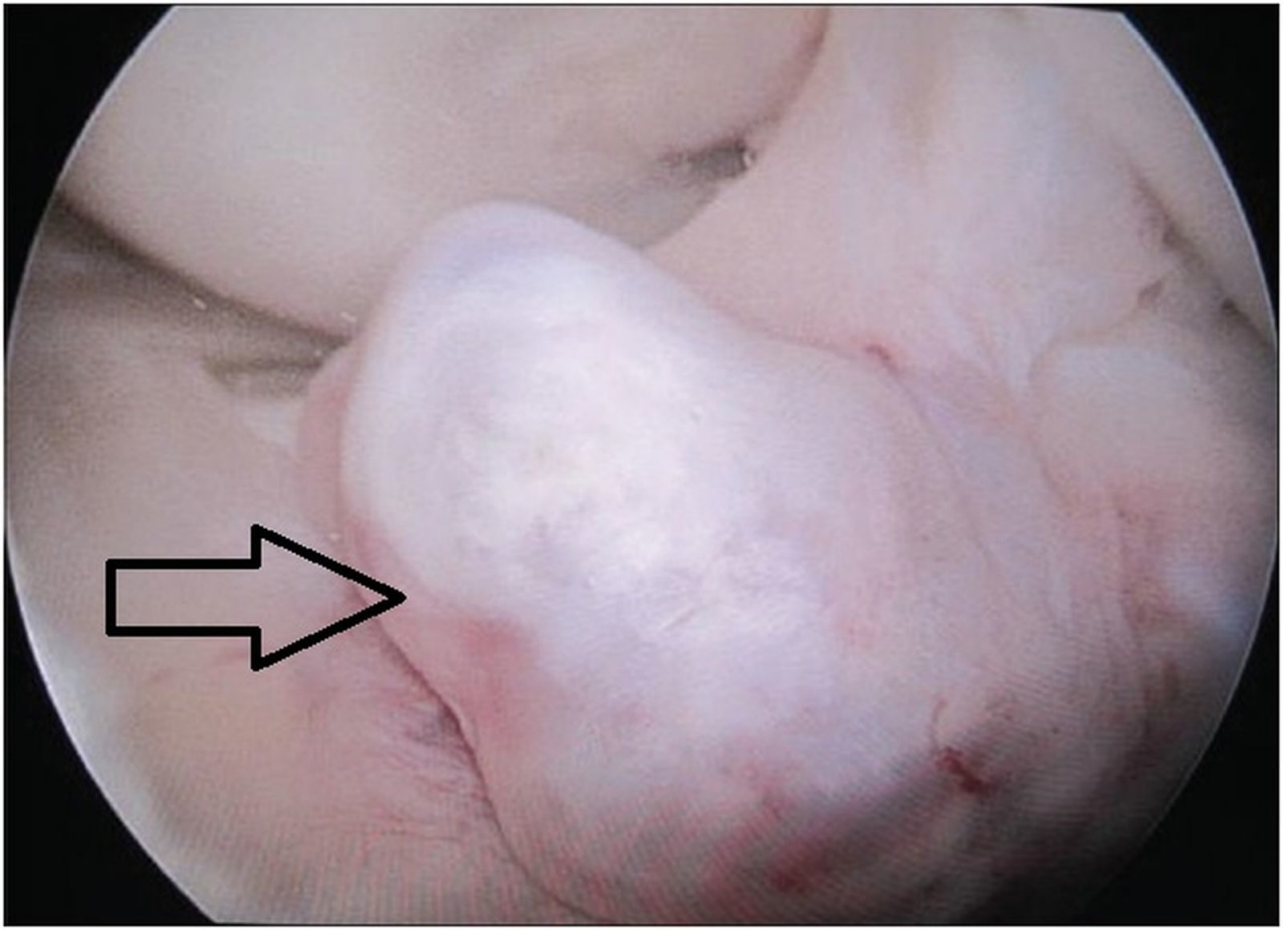
Intraoperative arthroscopic view showing ganglion cyst of ACL arising from tibial insertion ACL: anterior cruciate ligament

**Figure 4:**
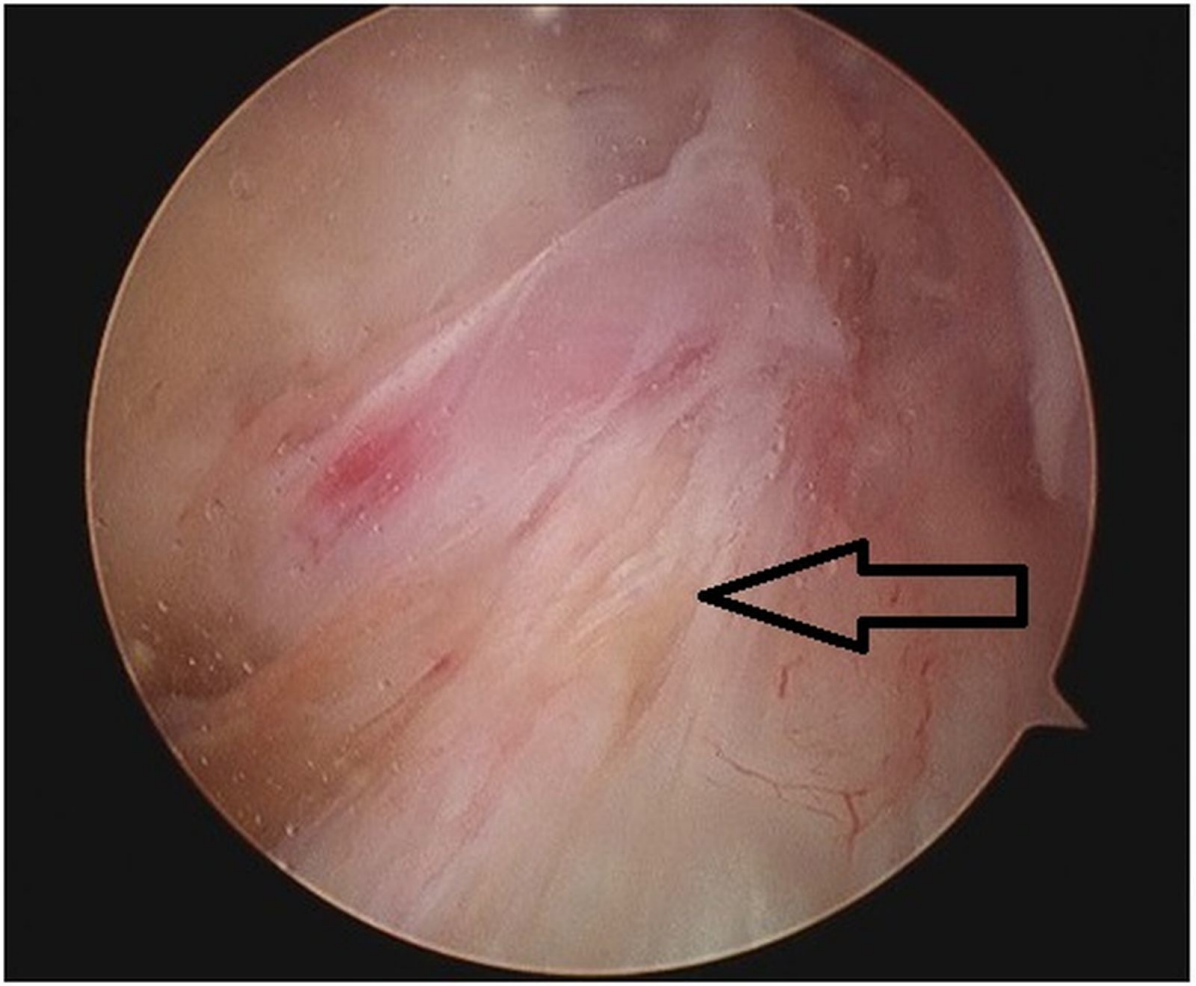
Intraoperative arthroscopic view showing ganglion cyst within the substance of ACL ACL: anterior cruciate ligament

Treatment modalities for ACL ganglion cyst include arthroscopic excision or puncture, CT scan, and ultrasound-guided aspiration [25-26]. ACL ganglion cyst at arthroscopy may appear as a cystic mass with defined margin on the ligament, or it may appear to be a substance with intact tibial and femoral insertion (Figures 3-4).

Arthroscopic decompression with debridement of the cyst is the treatment of choice for instant relief of pain, improvement in range of motion, and return to sporting activities. Arthroscopy affords complete excision of the cyst, along with diagnosis and treatment of other associated intra-articular knee derangements. No recurrence of symptoms or cyst on MRI post-arthroscopic excision has been reported in the literature; the longest follow-up was five years. Aspiration under CT scan and an ultrasound-guided approach have been reported with excellent results. It was reported to produce instant relief of pain and improvement in range of motion. However, there are concerns about the possibility of recurrence since it is impossible to completely excise the sac of the cyst, although no recurrence has been reported. The other drawbacks of this method are the inability to address associated intra-articular pathologies. This method may only be considered in select cases.

Anterior cruciate ligament mucoid degeneration

ACL mucoid degeneration is an uncommon pathological condition; the pathogenesis, prevalence, and association with other intra-articular knee structural damage are still poorly understood. It often presents with progressive knee pain, restriction in range of motion without a significant history of trauma, and without knee instability. ACL mucoid degeneration was first described in 1999 based on biopsy findings. It is characterized by degeneration of collagen fibers and deposition of new glycosaminoglycans [27]. With increasing use of MRI to evaluate the knee joint, ACL mucoid degeneration is increasingly being diagnosed incidentally when evaluating knees for pain and stiffness. The prevalence of ACL mucoid degeneration on MRIs of the knee in two large series was 0.42% and 4.30%, respectively. The median age was 43 years old (range: 22 - 66 years) according to Bregin’s study in Salvati, et al. [28]. However, Cha, et al. [29] reported a higher median age of 51 years (range: 35 - 75 years) in their review of 66 patients. Sex ratio was 1.28 females (F) to 1 male (M) according to Salvati, et al., contrary to Cha, et al. who showed a significant female preponderance of 4:1 (F:M). The pathogenesis of ACL mucoid degeneration is still unresolved, but injury, ganglion cyst, and the degenerative process leading to loss of the synovial lining of the ACL have been postulated [30]. It was also suggested that ACL degeneration in the young and athletic populations might be due to repeated microtrauma, while, in older patients, it could be due to progressive degenerative ACL lesion with a concomitant degenerative meniscal lesion.

Clinical Features

Insidious onset chronic knee pain behind the patella is the most common complaint [31-32]. The duration of symptoms is variable from weeks to months. The pain may limit the terminal movements of the knee. There is usually no antecedent history of significant trauma, and when present, it is normally trivial. Pain and limitation in range of motion have been attributed to both increased volume and tension within the ligament and mechanical impingement with unique function of the ACL in providing nociceptive sensory signals [33]. Locking and grinding sensations may be present. Pain and knee stiffness do not respond to non-steroidal anti-inflammatory drugs (NSAIDs) or physiotherapy. Clinical examination may show limitation of motion, joint line tenderness [34], joint effusion, and a positive grinding test of the meniscus. The Lachman test, anterior drawer test, and pivot shift test for ACL integrity are usually negative. These clinical features, however, are not pathognomonic for ACL mucoid degeneration, as they are common presentations of internal knee derangement; however, they should raise suspicion and the need for further evaluation with MRI (especially if the symptoms are nonspecific and unresponsive to non-steroidal anti-inflammatory drugs and physiotherapy).

Investigations

**Figure 5:**
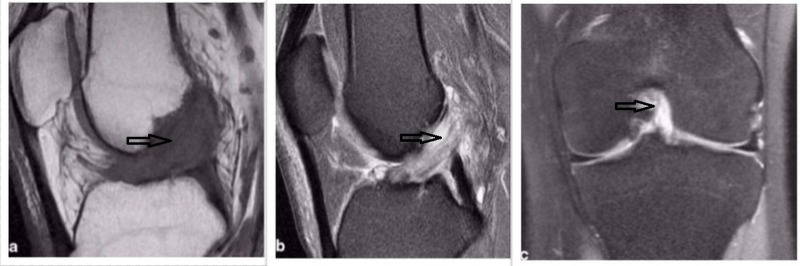
MRI showing ACL mucoid cyst a) T1-weighted sagittal view; b) T2- weighted sagittal view; c) T2- weighed coronal view MRI: magnetic resonance imaging; ACL: anterior cruciate ligament

Conventional x-rays do not have any specific role in the diagnosis of ACL mucoid degeneration but will reveal associated osteoarthritic changes, if present. MRI is the main stay of diagnostic imaging for mucoid degeneration. The MRI features suggestive of ACL mucoid degeneration are (a) abnormally thickened and an ill-defined, bulky ACL, (b) increased intra-ligamentous signals (intermediate signal intensity on T1-weighted images, high signal intensity on T2-weighted images, and proton density weighted images) on all sequences interspersed among visible intact fibers (the celery stalk appearance), and (c) maintenance of normal orientation and continuity of the ACL (Figure 5).

ACL mucoid degeneration coexists with ACL ganglion cyst, and there is a higher association of ACL mucoid degeneration with a meniscal tear, chondral damage, and intraosseous cyst at the femoral and tibial attachment of the ligament. ACL mucoid degeneration has been mistakenly reported as ACL rupture on MRI.

Treatment

**Figure 6:**
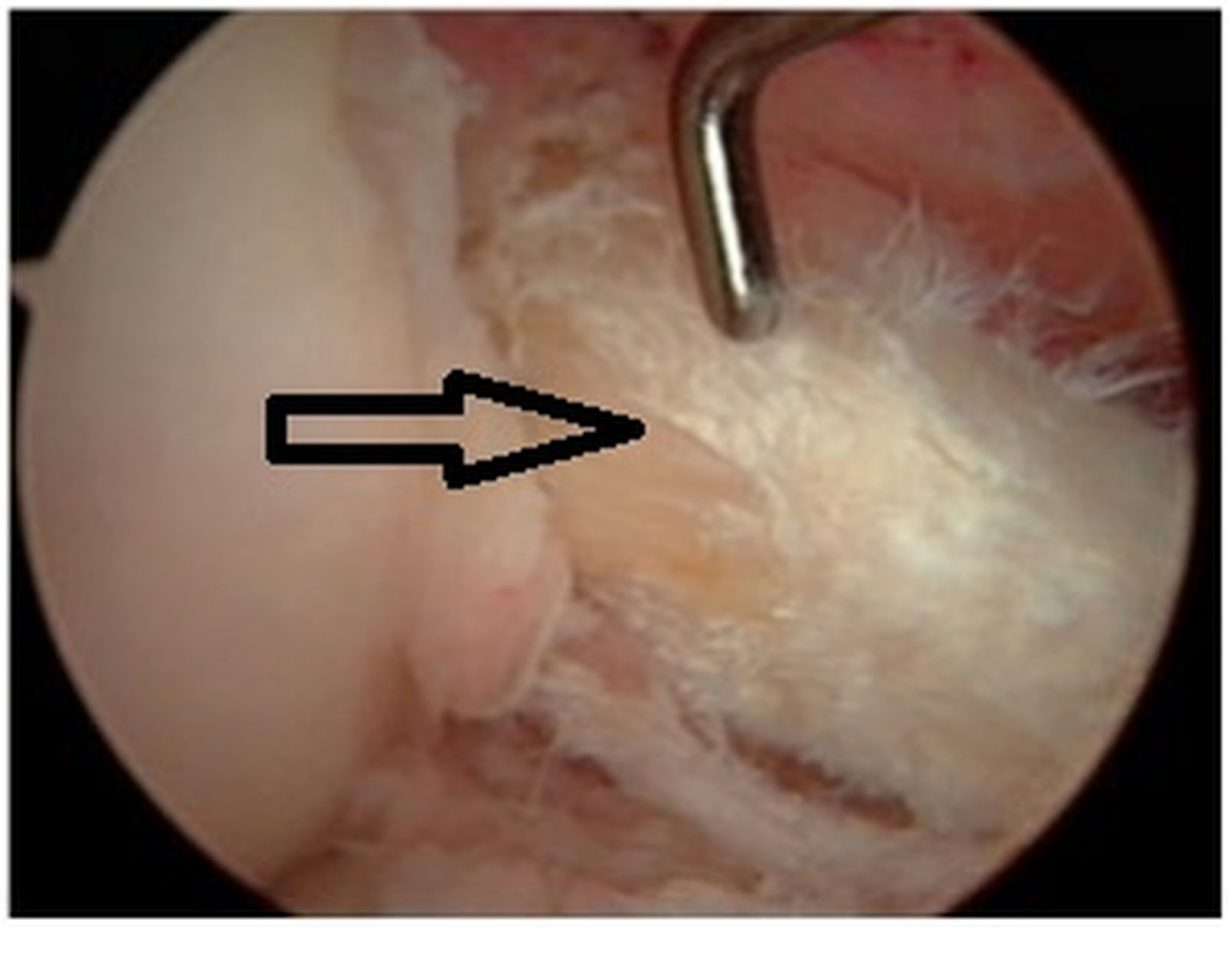
Intraoperative arthroscopic view of mucoid degeneration of ACL showing hypertrophied, intact fibers with absence of shining synovial lining ACL: anterior cruciate ligament

Pain and limitation in range of motion of the knee due to mucoid degeneration do not respond completely to NSAIDs and physiotherapy [35]. Unlike ACL ganglion cyst, where CT scan and ultrasound-guided cyst aspiration is an effective treatment option, the interstitial nature of mucoid degeneration precludes this method. Arthroscopic treatment with the aim of debulking the lesion without compromising the integrity of the ACL is the treatment of choice. On arthroscopy, the ACL mucoid cyst is viewed as homogenous and hypertrophied with increased diameter, intact and competent fibers with normal orientation, loss of shining synovial lining, the absence of ligamentous mucosa, and the flow of a yellow mucoid substance upon probing [36] (Figure 6).

Arthroscopic treatment consists of debridement and partial resection of the afflicted portion of the ACL, leaving intact the remnant of the anteromedial and posterolateral border and intact tibial and femoral attachment without impingement on the roof or lateral wall of the notch [37]. Partial resection of the ACL results in immediate relief of pain and improvement in range of motion. The patient can immediately start full-weight-bearing and return to activity very soon. Aggressive total resection of the entire lesion is not supported in the literature. There was no recorded case of recurrence after partial resection in the literature.

## Conclusions

Ganglion cyst and mucoid degeneration of the anterior cruciate ligament are the non-traumatic lesions that present with chronic knee pain and severe limitation in terminal range of motion of the knee. These are often of insidious onset, without any history of significant trauma, and they do not respond to conventional NSAIDs and physiotherapy. These conditions should be considered in the differential diagnosis of knee pain of unspecified origin. Magnetic resonance imaging is the gold standard for radiological diagnosis, and arthroscopic treatment results in the immediate resolution of symptoms without compromising the integrity of the ligament.

## References

[1] Fealy S, Kenter K, Dines JS (2001). Mucoid degeneration of the anterior cruciate ligament. Arthroscopy.

[2] Maikano A, Pascual-Garrido C, Rolon A (2011). Mucoid degeneration of the anterior cruciate ligament: MRI, clinical, intraoperative and histological findings. Knee Surg Sports Traumatol Arthrosc.

[3] Parish EN, Dixon P, Cross MJ. Ganglion (2005). Cyst of the anterior cruciate ligament: a series of 15 cases. Arthroscopy.

[4] Garcia-Alvarez F, Garcia-Pequerul JM, Avila JL (2000). Ganglion cyst associated with cruciate ligaments of the knee: a possible cause of recurrent knee pain. Acta Orthop Belg.

[5] Bergin D, Morrison WB, Carrimo JA (2014). Anterior cruciate ligament ganglia and mucoid degeneration: coexistence and clinical correlation. Am J Roentgenol.

[6] Lintz F, Pujol N, Boisrenoult P (2011). Anterior cruciate ligament mucoid degeneration: a review of literature and management guidelines. Knee Surg Sport Traumatol Arthrosc.

[7] Krudwig WK, Schute KK, Heinemann C (2004). Intra-articular ganglion cyst of the knee joint: a report of 85 cases and review of the literature. Knee Surg Sports Traumatol Arthros.

[8] Matrawy KA, El-Nekeidy AM, Al-Dawody A (2012). Mucoid degeneration of the anterior cruciate ligament: frequently under-diagnosed entity in MRI. Egypt J Radiol Nucl Med.

[9] Plotkein B, Agarwal VK, Varma R (2009). Ganglion cyst of the anterior cruciate ligament. Radiol Case Rep.

[10] Pedrinell B, Castellana FB, Fontes RB (2002). Anterior cruciate ligament: a case report. Sao Paulo Med J.

[11] Bui-Mansfield LT, Yougberg RA (1997). Intraarticular ganglia of the knee: prevalence, presentation,etiology and management. Am J Roentgenol.

[12] Willis-Owen CA, Konyves A, Martin DK (2010). Bilateral ganglion cyst of the cruciate ligament: a case report. J Orthop Surg (Hong Kong).

[13] Sayampanathan AA, Koh TH, Lee KH (2016). Anterior cruciate ligament ganglion causing flexion restriction: a case report and review of the literature. Ann Transl Med.

[14] Battagalia TC, Freilch AM, Diduh DR (2007). An intra–articular knee cyst in a two-year-old associated with an aberrant anterior cruciate ligament. Knee Surg Sports Traumatol Arthrosc.

[15] Kaatee R, Kjartansson O, Brekkan A (1994). Intra-articular ganglion between the cruciate ligament of the knee. A case report. Acta Radiol.

[16] Kakutanl K, Yoshiya S, Matsui N (2003). An intraligamentous ganglion cyst of anterior cruciate ligament after a traumatic event. Arthroscopy.

[17] Mao Y, Dong Q, Wang Yi (2012). Ganglion cyst of the cruciate ligament: a series of 31 cases and review of the literature. BMC Musculoskelet Disord.

[18] Lunhao B, Yu S, Jiashi W (2011). Diagnosis and treatment of ganglion cyst of the cruciate ligaments. Arch Orthop Trauma Surg.

[19] Sloane J, Gulati V, Penna S (2010). Large intrarticular anterior cruciate ligament ganglion cyst presenting with the inability to flex the knee. Case Rep Med.

[20] Rolf C, Waston TP (2006). Case report: intra-tendinous ganglion of the anterior cruciate ligament in a young footballer. J Orthop Surg Res.

[21] Krishnamurthy A, Soraganvi P, Kumar JM (2014). Ganglion cyst of the knee associated with anterior cruciate ligament: a report of three cases. Saudi J Sports Med.

[22] Mittal S, Singla A, Nag HL (2014). Dual ACL ganglion cyst: significance of detailed arthroscopy. Case Rep Orthop.

[23] Sumen Y, Ochi M, Deie M (1999). Ganglion cyst of the cruciate ligament detected by MRI. Int Orthop.

[24] Ryan RA, Munk PL (2004). Radiology for the surgeon: Musculoskeletal case 31. Anterior cruciate ligament cyst. Can J Surg.

[25] Campagnolo DI, Davis BA, Blacksin MF (1996). Computed tomography- guided aspiration of a ganglion cyst of anterior cruciate ligament: A case report. Arch Phys Med Rehabil.

[26] Sonnery-Cottet B, Guimarães TM, Daggett M (2016). Anterior cruciate ligament ganglion cyst treated under computed tomography guided aspiration in a professional soccer player. Orthop J Sports Med.

[27] Kwee RM, Ahlawat S, Kompel AJ (2015). Association of mucoid degeneration of anterior cruciate ligament with knee meniscal and cartilage damage. Osteoarthritis Cartilage.

[28] Salvati F, Rossi F, Limbucci N (2008). Mucoid metaplastic-degeneration of anterior cruciate ligament. J Sports Med Phys Fitness.

[29] Cha JR, Lee CC, Cho SD (2013). Symptomatic mucoid degeneration of the anterior cruciate ligament. Knee Surg Sports Traumatol Arthrosc.

[30] Chudasama CH, Chudasama VC, Prabhakar MM (2012). Arthrocopic management of mucoid degeneration of anterior cruciate ligament. Indian J Orthop.

[31] Narvekar A, Gajjar S (2004). Mucoid degeneration of anterior cruciate ligament. Arthroscopy.

[32] Kumar A, Bickerstaff DR, Grimwood JS (1999). Mucoid cystic degeneration of the cruciate ligament. J Bone Joint Surg Br.

[33] Kim TH, Lee DH, Lee SH (2008). Arthroscopic treatment of mucoid hypertrophy of the anterior cruciate ligament. Arthroscopy.

[34] Pandey V, Suman CPS, Sharma S (2014). Mucoid degeneration of anterior cruciate ligament: management and outcome. India J Orthop.

[35] Hensen JJ, Coerkamp EG, Bloem JJ (2007). Mucoid degeneration of anterior cruciate ligament. JBR-BRT.

[36] Lintz F, Pujol N, De Jour D (2010). Anterior cruciate ligament degeneration: selecting the best treatment option. Orthop Traumatol Surg Res.

[37] Khanna G, Sharma R, Bhardwaj A (2016). Mucoid degeneration of the anterior cruciate ligament: partial arthroscopic debridement and outcomes. J Arthr Joint Surg.

